# Progress in the Development of Universal Influenza Vaccines

**DOI:** 10.3390/v12091033

**Published:** 2020-09-17

**Authors:** Wenqiang Sun, Tingrong Luo, Wenjun Liu, Jing Li

**Affiliations:** 1 CAS Key Laboratory of Pathogenic Microbiology and Immunology, Institute of Microbiology, Chinese Academy of Sciences, Beijing 100101, China; sunwenqiangsyfq@163.com; 2 State Key Laboratory for Conservation and Utilization of Subtropical Agro-Bioresources & Laboratory of Animal Infectious Diseases, College of Animal Sciences and Veterinary Medicine, Guangxi University, Nanning 530004, China; tingrongluo@gxu.edu.cn; 3 University of the Chinese Academy of Sciences, Beijing 100049, China; 4 Institute of Microbiology, Center for Biosafety Mega-Science, Chinese Academy of Sciences, Beijing 100101, China

**Keywords:** influenza virus, antigenic variation, universal vaccine, cross-protection, cellular immunity

## Abstract

Influenza viruses pose a significant threat to human health. They are responsible for a large number of deaths annually and have a serious impact on the global economy. There are numerous influenza virus subtypes, antigenic variations occur continuously, and epidemic trends are difficult to predict—all of which lead to poor outcomes of routine vaccination against targeted strain subtypes. Therefore, the development of universal influenza vaccines still constitutes the ideal strategy for controlling influenza. This article reviews the progress in development of universal vaccines directed against the conserved regions of hemagglutinin (HA), neuraminidase (NA), and other structural proteins of influenza viruses using new technologies and strategies with the goals of enhancing our understanding of universal influenza vaccines and providing a reference for research into the exploitation of natural immunity against influenza viruses.

## 1. Introduction

Influenza viruses comprise enveloped RNA viruses with a genome composed of eight single-stranded negative-sense RNA fragments encoding polymerase subunits PA, PB1, and PB2, envelope proteins hemagglutinin (HA) and neuraminidase (NA), nucleoproteins (NP) binding to genomic RNA, matrix protein M1 and ion channel protein M2, nonstructural protein NS1, nuclear export protein NEP, and the more recently discovered PB1-F2, PB1 N40, PA-X, and M42 proteins [1,2,3,4,5]. Annual seasonal outbreaks of influenza occur in winter and spring, which can seriously threaten the life of individuals with increased susceptibility to influenza such as the elderly, children, and those with low immunity. According to the statistics of the U.S. Centers for Disease Control and Prevention (CDC), 39–56 million people were infected by influenza viruses from October 2019–April 2020, among whom 24,000–62,000 died (https://www.cdc.gov/flu). Moreover, based on statistics from the World Health Organization (WHO), 3–5 million cases of severe respiratory disease induced by influenza virus infection occur every year (https://www.who.int/).

Currently, vaccines are considered as the best choice for the prevention and control of influenza. At present, conventional commercial vaccines include whole inactivated influenza virus vaccines, influenza split vaccines, and attenuated vaccines. For whole inactivated influenza virus vaccines, the virus is inactivated while maintaining antigenicity allowing neutralizing antibodies to be produced following vaccination. However, these vaccines offer weak inter-subtype cross-protection and exhibit high incidence of fever upon use in children. Thus, they are not suitable for children under 12 years of age [6,7,8,9]. For influenza split-virus vaccines, the components of an inactivated virus are segregated using splitting agents, removing nucleic acids and macromolecular proteins while retaining only antigens HA and NA, matrix proteins, and nucleoproteins. Such vaccines are widely used. These vaccines have few side effects and high immunogenicity albeit weak inter-subtype cross-protection [10,11]. In turn, attenuated live vaccines are prepared using HA and NA from the epidemic strains recommended by the WHO in combination with cold-adapted influenza strains as the internal skeleton. These cold-adapted strains can replicate effectively at 25–33 °C although their replication is limited at 37 °C. This type of vaccine can be administered via nasal drops, with the limited replication of viruses in the upper respiratory tract able to stimulate the body to produce high-levels of sIgA and cellular immune response, thereby generating strong cross-protection [12,13,14,15]. However, a risk of gene reassortment exists between this attenuated live vaccine and wild strains. In addition, because of the constant reassortment and mutation of gene fragments in influenza viruses, multi-clade strains also frequently appear (e.g., H5 subtype has multiple clade: clade 2.3.4.4, clade 2.3.2.1, etc.), requiring the development of new targeted vaccines [16]. Thus, numerous challenges exist with regard to controlling the influenza virus using traditional influenza flu vaccines [17,18,19,20,21], highlighting the urgent need for development of universal influenza vaccines to promote influenza prevention and control.

At present, research on universal vaccines is mainly focused on the stem region of HA2, chimeric HA, M2e, NP, and T/B cell epitopes. In this review, the target virus proteins and immune effects of universal vaccines are reviewed in order to analyze the advantages and disadvantages of different universal vaccine strategies and provide a theoretical basis for developing safe, effective, and quality-controllable universal influenza vaccines.

## 2. Development of Universal Vaccines Targeting Influenza Virus Surface Proteins

### 2.1. Universal Vaccines Targeting the Stem Region of HA

**Figure 1:**
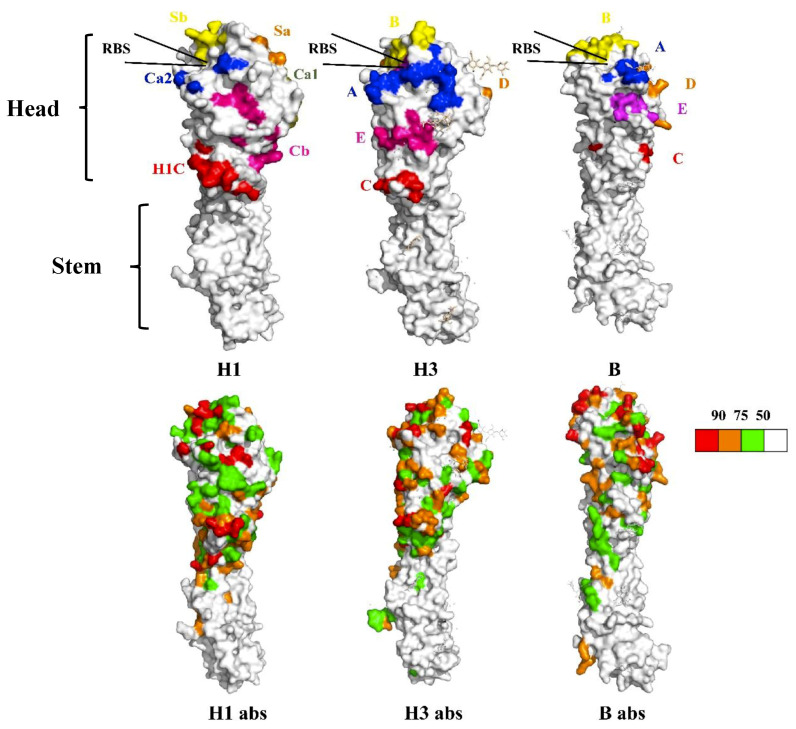
Mutation frequency of different antigenic regions and surface amino acids in the hemagglutinin (HA) protein of influenza viruses. H1 represents the 3D structure of A/Puerto Rico/8/34 (H1N1) HA protein (PDB ID: 1RU7) on which the location and distribution of different antigenic regions (Ca1, Ca2, Sa, Sb, Cb, and H1C) are indicated. H3 represents the 3D structure of A/X-31 H3 subtype HA protein (PBD ID: 2VIU) illustrating the location and distribution of different antigenic regions (A, B, C, D, and E). B represents the 3D structure of B/Lee/40 B subtype HA protein (PDB ID: 1RFT) highlighting the location and distribution of different antigenic regions (A, B, C, D, and E). H1 abs, H3 abs, and B abs illustrate the mutation frequency of surface amino acids on the respective HA proteins, with their color representing the intensity of mutation frequency based on H1N1 (*n* = 531, isolated between 1918–2008), H3N2 (*n* = 968, 1968–2005), and flu B (*n* = 209, 1940–2007).

The structure of HA is comprised of a globular head region and a stem region. In general, the HA head region is immuno-dominant, whereas the stem only provides a limited immunogenic subdominance following immunization with inactivated influenza virus vaccines. The neutralizing antibody against HA can effectively hinder the binding of the virus to the corresponding target cell receptor of host, thereby preventing influenza virus entry. However, due to the high frequency of antigenic drift and antigenic shift, the HA head—especially the receptor binding site (RBS)—of influenza viruses varies considerably between different subtypes; thus, it is difficult to utilize this region in the development of universal vaccines. In comparison, the low mutation frequency of HA protein surface amino acids indicates that the stem region is relatively conserved among different HA subtypes (Figure 1). Therefore, “stem only” HA, also termed “headless” HA, is considered as a promising candidate for the development of universal vaccines [22]. The principle of this strategy is that the stem region of HA will be better exposed spatially when the HA head is completely deleted, thereby significantly enhancing the immunogenicity of the stem. Consequently, the HA stem will induce protective neutralizing and non-neutralizing antibodies, which then activate antibody-dependent cellular cytotoxicity and phagocytosis [23]. In addition, the HA stem also activates T-cell immunity, producing killer CD8^+^ T cells to kill infected cells [24].

**Figure 2:**
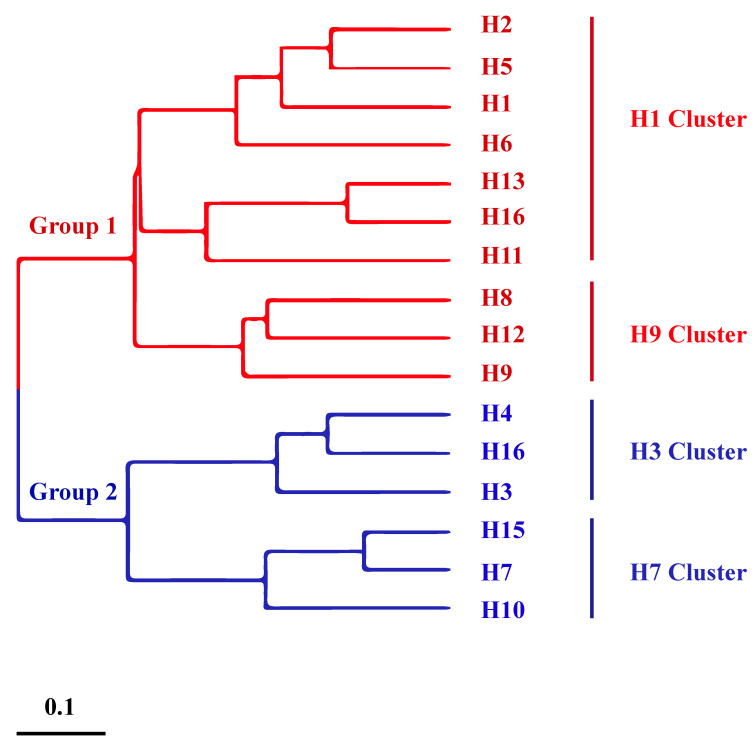
Phylogenetic tree of HAs of different subtypes (H1–H16) of influenza viruses. The phylogenetic tree was constructed using the neighbor-joining (NJ) method within MEGA software (version 7.0). The colors of the trees are edited using Adobe Illustrator software. The scale bar indicates the average number of amino acid substitutions per site.

Notably, research to develop an influenza headless HA universal vaccine has been continuous over the past decade. Steel et al. [25] designed a virus-like particle vaccine expressing PR8 (H1N1) and HK68 (H3N2) headless HA proteins. Vaccination with the headless HA provided full protection against death and partial protection against disease following challenge with the lethal PR8 virus in mice and elicited immune sera with broader reactivity (as tested by enzyme-linked immunosorbent assay—ELISA) than that obtained from mice immunized with full-length HA [25]. Similarly, Wohlbold et al. [26] expressed soluble PR8 headless HA using insect cells. This could induce completely homologous and partial heterosubtypic protection against challenge with H1N1, H5N1, and H6N1 strains in vaccinated mice [26]. Yassine et al. and Impagliazzo et al. independently developed a stable full-length and a truncated HA stem trimer, respectively, based on influenza A virus HA. Studies in animals demonstrated that the stable full-length HA stem trimer could completely protect against homologous strains and heterologous H5N1 strains, whereas the stem-truncated vaccine could only protect against 60% of subtype H5N1 [27,28]. Several other studies in animals have also shown that most “headless” stem vaccines can provide complete protection against homologous viruses albeit only partial protection against heterologous viruses [29,30,31]. In general, the protection rate of HA stem vaccines differs according to the variance between different HA groups (Figure 2). Thus, the potential exists for the development of semi-universal vaccines for different subtypes of influenza viruses in the same group based on the shared stem region of HA.

Furthermore, it is worth noting that as HA is a glycoprotein, whether the “headless” HA protein can be properly glycosylated also exerts key influence on the immune effects of the vaccine. Studies have shown that glycosylation in the HA stem affects the protective effect of influenza vaccines. For example, after the glycosylation site of influenza viruses in Group 2 was introduced to HA of H1N1 (Group 1), the associated vaccine afforded resistance to the challenge of viruses in Group 2 following immunization, but lost its protection against the H5N1 strain in Group 1 [32]. Therefore, HA stem protein sequences encompassing the most reasonable allocation of glycosylation sites can be designed to optimize immunogenicity by studying the influence of glycosylation sites on the immune effect of the vaccine. Additionally, choosing a eukaryotic expression system with modified functions rather than a prokaryotic expression system to express headless HA also constitutes a key factor promoting the success of developing HA universal vaccines, with the best option being a mammalian protein expression system [33].

Therefore, overall, universal vaccines based on the stem region of HA exhibit good homologous protection but unfortunately cannot provide complete or effective protection against heterologous (especially different HA group) viruses. Choosing the HA stem region of one subtype as a vaccine is unlikely to produce complete protection against other subtypes. Rather, it remains necessary to explore a more conserved region or chimeric HA stem (Section 2.2) that reflects two HA groups to develop universal vaccines targeting the HA stem region.

### 2.2. Chimeric HA Universal Vaccines

Chimeric HA consists of a highly variable HA head region—derived from different subtypes of influenza viruses—and a conservative HA stem region, with H1, H3, and B influenza having been reported [34,35,36]. Different globular heads produce different chimeric HAs (cHAs). A broad-spectrum immune response can be induced by continuously immunizing multiple cHA proteins or chimeric influenza viruses rescued by reverse genetic technology. The objective of this strategy is to strengthen the body’s immune memory of the HA stem by continuously immunizing with vaccines against the same HA stem but different subtypes of HA heads. For example, Krammer et al. [24] replaced the head region of HA1 and the stem region of HA2 of different subtypes in Group 1 and sequentially immunized against the different chimeric HA proteins. However, the resulting chimeric HA vaccines could provide protection for the virus in Group 1 but could not provide complete protection for the virus in Group 2 [24]. Liu et al. [37] replaced the head region of the pH1N1 strain with that of the H5, H8, or H9 subtypes. Following rescue of the chimeric HA attenuated virus, ferrets were immunized using two methods: Attenuation-inactivation and attenuation-attenuation of the chimeric HA virus. The attenuation-attenuation immunization strategy produced an extensive cross-antibody response specific to the stem region and also induced CD4^+^, IFN-γ, and CD8^+^ IFN-γ-specific effector T cells against the stem region of HA in the peripheral blood. Moreover, the virus load of each organ was lowest in the pH1N1 and H6N1 virus-infected animals immunized by attenuation-attenuation [37]. Nevertheless, this experiment did not incorporate multiple lethal homologous viruses to challenge and was therefore unable to ascertain whether the vaccine could produce protection against death.

In recent years, the development of chimeric HA vaccines has also incorporated the NP and M1 proteins of influenza viruses. Additionally, immunization has been carried out in the form of a DNA vaccine or virus vector vaccine, which yields extensive protection. Specifically, the results revealed that vaccines expressing both sets of antigens provided enhanced protection against influenza virus challenge when compared to that from vaccination with vaccines expressing only one set of antigens [36,38,39]. Nevertheless, to facilitate further development of chimeric universal vaccines problems such as the requirement for multiple immunizations and the immunodominance of the HA head region over the HA stem region need to be resolved.

### 2.3. NA Universal Vaccines

NA constitutes a glycoprotein located in the envelope of influenza viruses that help mature virus particles separate from infected host cells [40]. Notably, influenza can be effectively treated by inhibiting the activity of NA. At present, commercially available neuraminidase inhibitors, such as zanamivir and oseltamivir, can effectively block the replication of influenza viruses. However, owing to the variation in influenza viruses, the antigenicity of NA is changing and gradually producing drug resistance [41,42]. Because of these changes in antigenicity, anti-NA drugs no longer play an effective antiviral role; thus, it is necessary to develop novel influenza vaccines. Toward this end, NA protein represents an excellent candidate target protein for influenza vaccines from the perspective of immunogenicity. Lampejo et al. [43] expressed NA proteins of different subtypes (type A influenza N1–N9; Ya88 B type influenza NA) using a baculovirus expression system. The immunization of mice was shown to provide protection against infection with homologous NA subtype viruses. Specifically, the type A influenza NA vaccine provided almost no protection against infection with heterologous NA subtype influenza viruses but afforded strong cross-protection for influenza viruses with the same NA subtype and different HA subtypes. In comparison, the Ya88 B vaccine provided complete protection against heterologous Victoria influenza virus infection [43]. Other studies have shown that monoclonal antibodies of NA isolated from H3N2 influenza virus-infected donors bind with exceptional breadth to multiple different influenza A and B virus NAs. These antibodies neutralize the virus, mediate effector functions, are broadly protective in vivo, and inhibit NA activity by directly binding to the active site [44]. Another study showed that following immunization with seasonal inactivated influenza vaccines (IAV)–Sanofi Pasteur Fluzone; A/California/07/2009X-179A (H1N1) pdm09, A/Texas/50/2012 X-223A (H3N2), B/Massachusetts/2/2012, and B/Brisbane/60/2008 viruses—mice express monoclonal antibodies with broad and potent antiviral activity against both IBV Victoria and Yamagata lineages, affording both prophylactic and therapeutic activity [45]. These results suggest that the same neutralization epitope exists between different subtypes of NA and that obtaining the conserved neutralization epitopes of NA of different subtypes may be the key to producing NA universal vaccines.

In summary, a universal NA vaccine strategy could be expected to develop extensive protection against the same NA of different HA strains. Nevertheless, no NA universal vaccines have been tested in humans until to now. Therefore, there are still many challenges to develop a universal influenza vaccine for NA.

## 3. Universal Influenza Vaccines Targeting Other Structural Proteins of Influenza Viruses

Along with the use of the two external antigen proteins—HA and NA—the use of relatively conservative internal structural proteins to develop universal influenza vaccines has also become a research focus. To date, the M2e, M1, and NP proteins have been the most studied. The mechanisms by which they produce protective immunity are as follows. M2e constitutes the extracellular region of M2 protein responsible for controlling ion channels, with antibodies against this region able to block this function. In turn, the relatively conserved M1 and NP protein sequences contain numerous conserved T cell epitopes, which can trigger the production of a broad-spectrum T cell response following immunization [39,46,47,48]. In particular, immunization of animals with an M2e vaccine comprised of multiple M2e proteins or M2e from different subtypes of influenza in series fused with other flagellins, epitopes, and adjuvants afforded good protective effects against infection with different influenza subtypes. Moreover, the differences in M2e amino acid sequence resulted in different vaccine protection levels [49,50,51,52]. To date, however, no M2e protein subunit has been tested singly in humans as a vaccine antigen.

In addition, the NP protein is relatively conserved among different subtypes and is therefore expected to extensively protect against infection with different subtypes. Lee et al. [53,54] expressed the NP protein of influenza B virus (B/Yamaga/16/1988 Yamagata lineage and B/Shangdong/7/1997 Victoria lineage) using recombinant adenovirus vectors to immunize animals by the intranasal route, demonstrating that both adenoviruses could produce NP-specific humoral immunity and CD8^+^ T cell immunity together with complete cross protection against the two lineages of influenza B virus. Moreover, they also demonstrated that the NP adenovirus vaccine exhibits greater efficacy when administered via an intranasal rather than intramuscular immunization route [53,54]. In addition, a team at the University of Oxford constructed a replication-deficient chimpanzee adenovirus-vectored vaccine expressing the conserved influenza antigens NP and M1, termed ChAdOx1 NP+M1 [55]. Clinical assessment demonstrated ChAdOx1 NP+M1 to be safe and immunogenic [55]. The team has also developed a new strategy by which heterologous two-dose vaccination with simian adenovirus and poxvirus vectors elicits long-lasting cellular immunity against influenza virus A in healthy adults [56]. Recently, they showed that vaccination with viral vectors expressing chimeric HA, NP, and M1 antigens could protect ferrets against H3N2 influenza virus infection [38]. Additionally, the data also showed that vaccination with the bivalent vectors described here would most likely induce both robust anti-HA stalk antibodies and long-lived T cell responses in humans [38]. In turn, immunization with OVX836, a recombinant protein candidate vaccine developed by Osivax consisting of the fusion of OVX313—a stable heptameric oligomerization domain—with the NP of influenza A (H1N1/WSN/1933) could induce strong NP-specific CD4^+^ and CD8^+^ T-cell systemic responses and establish CD8^+^ tissue memory T cells in the lung parenchyma in mice and protect mice against viral challenge with three different influenza A subtypes [57]. Notably, Osivax is conducting phase 1 clinical trials and will reportedly perform a more detailed immunologic assessment of OVX836 including its effect on cellular immune, CD4^+^, and CD8^+^ T-cell response (https://www.clinicaltrials.gov).

Moreover, some studies have designed universal vaccines by combining M2e and NP proteins and expressing the NP protein of influenza A virus in tandem with one or more M2e domains using adenovirus vectors. Both humoral and cellular immune responses were produced following immunization with these vaccines. However, only immunization with vaccines supporting the expression of NP and 4× M2e fusion in tandem induced elevated levels of IL-2 and IL-10 and strong cross-immunoprotection. These could protect against the challenge of homologous influenza A virus but not that of heterologous influenza B virus [58,59]. Others have constructed MVA-NP+M1 vaccines consisting of a replication deficient MVA viral vector expressing the NP and M1 antigens from the influenza A virus (H3N2, A/Panama/2007/99) [60]. In a phase I study, the safety and immunogenicity of MVA-NP+M1 were evaluated in six healthy adult participants, revealing that the vaccine is well-tolerated with only mild to moderate adverse events and induces significant vaccine-specific T cell responses by seven days following immunization [60]. A phase IIb study is also planned to assess immunogenicity and additional protective efficacy in older adults [61].

Together, these results support that M1, M2e, and NP proteins constitute effective targets for the development of universal vaccines. However, the constant gene mutation occurring in influenza viruses suggests the likelihood of changes in M2e amino acids, with even minimal alterations being likely to alter the antigenicity of the M2e protein owing to the short sequence of the M2e subunits. Therefore, targeting the M2e protein alone is not sufficient to develop a universal influenza vaccine. In the future, the differences between different subtypes of M2e will need to be resolved and M1 and NP protein sequences containing all the dominant T epitopes of different influenza virus subtypes will need to be screened out to allow development of a universal vaccine.

## 4. Novel Universal Influenza Vaccines

### 4.1. Universal Influenza Vaccines Targeting T/B Cell Epitopes

Epitope vaccines comprise a novel type of vaccine in which T/B cell epitopes are predicted using bioinformatics. The epitope peptides that stimulate the response of T/B cells are screened and obtained, and epitope vaccines are developed through viral vectors, fusion protein, or tandem expression. However, for influenza viruses, the sequence differences among different subtypes are so large that it is difficult to produce extensive protection through use of individual subtype strains. Therefore, the exploration of conservative or differential dominant T/B cell epitopes among different subtypes and their subsequent use to develop universal vaccines by means of expressing all the dominant T/B cell peptides may serve to produce excellent protection. Numerous attempts have been made to develop such influenza epitope vaccines. For example, the overlapping MHC class II restricted B cell epitopes and MHC class I restricted T cell epitopes have been screened out from the HA proteins of H1, H2, H3, H5, H7, and H9 subtypes and connected with other epitopes in series to construct plasmid DNA and virus vector vaccines. However, studies in animals found that the epitope vaccine group could produce extensive specific cell response but could not provide complete protection [62,63,64].

In addition, researchers have engineered conserved T cell epitopes of M1, NS1, PB1, and PA proteins, or M2e, HA fusion peptide, NP T cell epitopes, and the HA α-helical region into vaccinia virus vector-based vaccines for immunizing animals, which were challenged with A/WSN/33, A/PR/8/34 or A/California/07/09 strains of influenza virus [48]. However, the vaccine only delayed death and did not provide protection [48]. Hassan et al. [64] generated human adenovirus replication defective vectors to express multi-epitopes—M2e, HA fusion domain (HFD), T-cell epitope of nucleoprotein (TNP), and HA α-helix domain (HαD)—of an H5N1 avian influenza virus and evaluated its immunogenicity and protective efficacy in a mouse model. Immunized animals were then challenged with H5N2, H7N9, or H9N2 influenza virus. The epitope vaccine induced humoral and cell-mediated immune response and significantly reduced the viral load in mouse lung. Compared with the single HA subunit vaccine group, the viral load of each organ in the epitope vaccine group was lower and the protection was more extensive; however, the vaccine’s ability to protect animals was not mentioned [64]. In turn, Eickhoff et al. [63] used state-of-the-art immunoinformatic tools to identify putative pan-HLA-DR and HLA-A2 supertype-restricted T cell epitopes highly conserved among >50 widely diverse influenza A strains—representing hemagglutinin types 1, 2, 3, 5, 7 and 9–and constructed these epitopes into eukaryotic expression plasmids. The results showed that pan-HLA-DR epitope and HLA-A2 epitope vaccines all protected 50% of immunized animals from A/H3N2 or A/PR8/H1N1 influenza infection and significantly reduced the viral load of H3N2 infection [63].

Overall, these results suggest that current epitope vaccines are only capable of producing partial protection for immunized animals rather than serving as universal vaccines. Nevertheless, epitope vaccines offer unique advantages as they stimulate the wide range of the body’s immune response, are safe, and afford simple and rapid mass production, in accordance with the preferred direction of future universal influenza vaccine development. Notably, several epitope vaccines are undergoing clinical review. Multimmer-001 (M-001)—developed by BiondVax Pharmaceuticals Ltd. (Israel)—is a single recombinant protein containing nine conserved epitopes from the HA (four B and one Th epitopes), NP (two CTL and one Th epitope), and M1 (one peptide that contains both B and CTL epitopes) proteins of both influenza type A and type B strains (Victoria and Yamagata lineages) that are known to induce both humoral and cellular immunity. Currently, the M-001 vaccine is in phase IIb clinical trials to evaluate its safety and immunogenicity as a standalone vaccine or as a primer to an H5N1 influenza vaccine product (Fluart Innovative Vaccines Ltd., Hungary) in healthy adults (aged 18–60 years) [65,66,67,68]. In turn, FLU-v, developed by PepTcell (SEEK), is a peptide vaccine comprised of four synthetic peptides with conserved epitopes from influenza A and B strains designed to provide a broadly protective cellular immune response against influenza A and B. Results from phase IIb clinical trials showed that adjuvanted FLU-v recipients (*n* = 40) were significantly less likely to develop mild to moderate influenza disease (MMID) following intranasal challenge of A/CA04/H1N1 vs. placebo (*n* = 42) (32.5 vs. 54.8% *p* = 0.035) [69,70,71]. Nevertheless, overall the vaccine protection effect was poor. It is thus expected that considerable time will be required to develop a peptide vaccine. In addition, the diverse alleles of HLA-I and HLA-II in the population should be carefully considered in the development of universal influenza epitope vaccines.

### 4.2. Universal Mosaic Influenza Vaccines

Mosaic vaccines are a new vaccine strategy developed mainly for viruses with easily mutated genes and numerous subtypes. In these vaccines, the most conservative T cell epitopes are selected from viral gene sequences using bioinformatics to calculate and synthesize a chimeric gene sequence, and a Mosaic protein sequence covering potential T/B cell epitopes is then obtained using a specific genetic algorithm. Currently, Mosaic vaccines for acquired immunodeficiency syndrome (AIDS) prevention have entered phase III clinical trials [72,73,74]. Similarly, Mosaic vaccines offer good prospects for the prevention of influenza. In particular, studies of Mosaic H5 HA in animals show that this vaccine can elicit full protection against clade 0, 1, and 2 avian influenza viruses of H5N1 and also protect against seasonal H1N1 virus (A/Puerto Rico/8/34) [75,76,77]. In addition, Corder et al. [78] designed Mosaic H1 HA vaccines according to human H1 influenza HA sequences isolated from 1918 to 2018. Mice immunized with a prime or prime-boost strategy using recombinant adenovirus Ad5-mosaic were completely protected against A/Nanchang/1/99, A/FM/1/47, and A/PR/8/34 viruses. However, the study did not evaluate the vaccine’s protective effects against heterologous virus [78]. Nevertheless, because of the large sequence differences among different subtypes and the challenges associated with exploiting the huge sequence information base, it is expected that Mosaic vaccines will likely be limited to being designed using HAs of the same subtype, for which they produce good protective effects [78,79,80]. Accordingly, current Mosaic vaccines are based on the prediction of T/B cell epitopes as the main core technology in combination with a specific algorithm to screen out sequences with high T/B epitope coverage rate in the sequence database [75,79,80]. However, as variations are always present, it is difficult to obtain epitope sequences covering all subtypes while maintaining the correct HA protein conformation. Thus, current Mosaic vaccine strategies appear in direct contrast to those needed to develop a universal influenza vaccine that produces protection against heterogeneous virus. In the future, it may be warranted to develop Mosaic vaccines rather by continuously optimizing the algorithm based on different target proteins and producing sequences with a higher epitope coverage rate based on the principle of seeking common ground while preserving differences.

### 4.3. Nanoparticle Universal Influenza Vaccines

Nano-vaccine technology can package virus particles or effective antigens into virus-like nanoparticles. The resultant nanoparticle vaccine display antigens on the surface of particles or enclose antigens in the particles in order to increase the antigenicity and immunogenicity of the vaccine. At present, research on nanoparticle universal influenza vaccines is mainly focused on virus-targeted proteins—e.g., HA, HA stem, M2e, NP, NA, and mosaic—which are synthesized into nanoparticles to increase the immune effect of the vaccine [81,82,83,84,85,86,87].

In general, two strategies exist to increase the antigenicity of nanoparticle vaccines. The first is to display antigens on the surface of particles such as nano gold or silver, polymers, and other inorganic matter or on self-assembled ferritin, VLPs, chitosan, and other organic matter to improve the ability of immune cells to recognize the antigen. Single or multiple antigens are loaded onto the surface of the nanoparticles or fused and expressed with self-assembled proteins to form nanoparticles [83,85,88,89,90]. Kanekiyo et al. [91] combined the expression of the HA receptor binding domain (RBD) of the H1N1 subtype of two viruses fused with a ferritin nanoparticle scaffold sequence to assemble double-loaded nanoparticle vaccines. The immunization results showed that the double-load assembled nanoparticle vaccine could induce extensive humoral immunity, with levels of neutralizing antibodies that were higher than those of a single-assembly nanoparticle vaccine [91]. Nevertheless, this study did not provide vaccine protection data against homologous or heterologous virus in animals. Bernasconi et al. [92] loaded an HA trimer (A/Puerto Rico/8/34 H1N1) and 3M2e-3Eα (A/Victoria/3/75 H3N2) fusion protein onto the surface of nanoparticles to immunize animals using polysaccharides and palmitate liposomes as the core of the nanoparticles. The vaccine could induce immune protection, which was mediated by enhancing the level of CD4^+^ T cells in the lung and serum IgG and local IgA antibodies against HA and M2e. Notably, double-load M2e-HA nanoparticles produced complete protection against PR8 virus and high dose of single 3M2e-3Eα nanoparticles could produce complete protection against mouse-adapted X47 virus—a reassortant between A/Victoria/3/75 (H3N2) and A/Puerto Rico/8/34 (H1N1) [92]. However, this study also did not demonstrate vaccine protection data against heterologous virus in animals. Overall, these results suggest that nanoparticles are advantageous for small immunogenic proteins or small-molecule epitopes to enhance immunogenicity as well as for loading multiple proteins.

The second method is to encapsulate single or multiple antigens in particles using liposomes and then transport them into cells via the endocytosis mechanism of cells using a double-layer structure of lipids as a carrier, thereby enhancing the efficiency of cell processing and antigen presentation. Toward this end, researchers immunized animals with M2e of multiple influenza viruses encapsulated by liposome nanoparticles, which produced 90–100% survival following lethal challenge with H1N1 (A/PR/8/34) [93]. Dhakal et al. [94] selected ten highly conserved and well-characterized influenza A virus chemically synthesized peptides (from M2e, HA, NP, PB1, NA, PA) and encapsulated these in liposomes. Following immunization, the peptide flu vaccine partially protected pigs from flu-induced fever and pneumonic lesions and reduced nasal virus shedding and viral load in the lungs [94]. In turn, Wang et al. [95] used pulmonary surfactant-biomimetic nanoparticles to package cGAMP as an adjuvant to mix with inactivated H1N1 influenza vaccine and then administered the immunization through the respiratory tract, resulting in extensive protection against H1NI, H3N2, H5N1, and H7N9 virus infection. As research on the protective mechanism of vaccines has demonstrated that lipid bilayer nanoparticles with a negative charge can effectively mediate endocytosis after binding with pulmonary surfactant protein in the alveoli, cGAMP could effectively activate the downstream STING pathway and stimulate the pulmonary epithelial cells to secrete cytokines, thereby enhancing the immune response of the body to T/B cells produced by vaccines. Notably, CD8^+^ T cells were found to play a key role in the cross-immunoprotection mediated by the vaccine [95]. Therefore, for some adjuvants or antigens that play a direct role in cells, lipid encapsulation might support effective transmission into cells through the endocytosis mechanism in order to enhance the immune effect of the vaccine.

**Figure 3:**
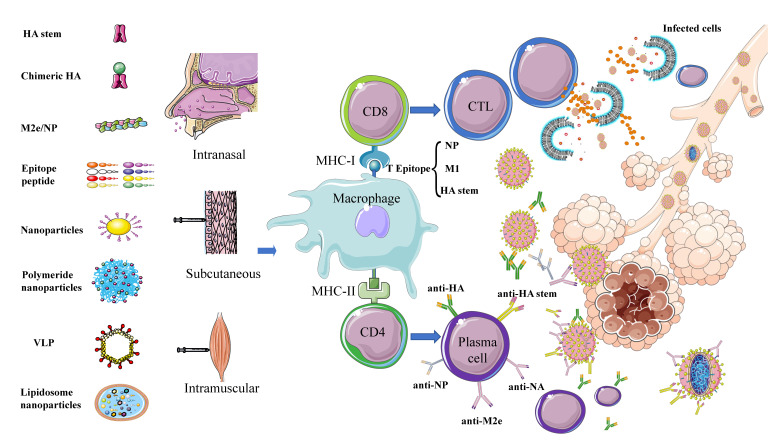
Schematic diagram of immune responses activated by different types of potential universal influenza vaccines. Universal influenza vaccines developed using different strategies involving different target proteins are administered by subcutaneous, intranasal, and intramuscular routes. The antigen is phagocytosed and processed by macrophages and other APC cells. Subsequently, B cell epitopes form a complex with MHC-II and are presented to the cell surface. Under the combined action of CD4 cells, B cells are activated to differentiate into plasma cells and secrete antibodies—e.g., anti-HA, anti-NA, anti-NP, anti-M2e, and anti-HA stem–to neutralize the virus. T cell epitopes—mainly, NP, M1, and HA stem—form a complex with MHC-I and are presented to the cell surface, under the action of CD8 cells and activate T cells to differentiate into CTLs to kill virus-infected cells.

Multiple key target proteins for universal vaccines have been identified in preliminary research (Figure 3). Together, the findings indicate that nanoparticles are suitable as carriers to transfer these antigens into cells or to be recognized and processed by the body, thereby effectively stimulating immune response to produce extensive immune protection.

## 5. Adjuvants

Adjuvants are key components in the study of universal influenza vaccines because of their potential to increase the titer and/or breadth of the antibody repertoire and enhance T cell immune responses, particularly subunit and inactivated vaccines. Adjuvants are injected into the body together with antigens to enhance the immune response ability of the body to antigens. The mechanism of action of adjuvants may rely upon a combination of various mechanisms including formation of a depot, induction of cytokines and chemokines, recruitment of immune cells, enhancement of antigen uptake and presentation, and promotion of antigen transport to draining lymph nodes [96,97]. Adjuvants include interferon pathway activators Poly I:C and cGAMP, cytokines including interferon and interleukin, and bacterial structural components flagellin and lipopolysaccharide (LPS) along with immunoregulatory oligonucleotide sCPG and synthetic chemical substances that play a role in immunological enhancement [51,95,98,99,100,101,102,103,104]. However, as universal influenza vaccines have been developed into multiple vaccine types—e.g., viral vectors, DNA, and subunit—to express influenza cocktail proteins such as HA stem, chimeric HA, M1, M2e, NP and epitope peptide (Figure 3), suitable adjuvants should be selected according to the vaccine strategy and type of desired immune response activation. For example, for universal influenza vaccines that mainly activate T cell response, such as HA stem, M1, NP protein, and T epitope vaccines, an adjuvant that activates the T cell response should be selected—e.g., Poly I:C, CAF01, CD1d ligands, AS01, or CpG-ODN. For vaccines that mainly activate humoral immune responses, such as those based on chimeric HA and M2e proteins, an adjuvant that activates antibody responses should be selected—e.g., aluminum adjuvant, MF59, or AS30 [105]. In summary, the scale and breadth of the antibody immune response and T cell immune response determine the ability of the vaccine to protect against heterologous strains. Choosing the right adjuvant can greatly improve the efficacy of the vaccine.

## 6. Perspective

The specific segmental gene structure and lack of polymerase proof reading function of influenza viruses are conducive to gene reassortment and mutation during infection of the host. Consequently, the development of vaccines always lags behind the evolution of variation in viruses. However, the development of universal influenza vaccines represents the key to preventing influenza. To date, most studies have focused on humoral and cellular immune responses, which can provide extensive protection after animals are immunized with the stem region of HA, chimeric HA, NA, and M2e, M1, NP, and T/B epitopes, Mosaic, or Nanoparticles (Figure 3). Nevertheless, although numerous achievements have been made, disadvantages as well as advantages of each method remain (Table 1). Accordingly, a universal vaccine should be designed to stimulate both humoral and cellular immunity, with the ability to activate CD8^+^ T cells being key to generating broad protection. In fact, the protective effects of many universal influenza vaccines have been evaluated only in animals and have not yet entered clinical trials in humans. There are a number of factors that prevent vaccines from being evaluated from animals to humans. The most important factor is that the evaluation of human clinical trials is different from that of animal trials, and there are very complex factors (infants, the elderly, pregnant women, sub-healthy populations and overweight/obese individuals) that often fail to achieve the same immune effects as animal trials. The huge cost of human clinical evaluation of vaccines is also one of the important reasons. Therefore, the prevention and control of influenza viruses is a common problem faced by mankind all over the world, which needs the joint efforts of every researcher.

## Figures and Tables

**Table 1:** Advantages and disadvantages of universal influenza vaccines.

Vaccine Type	Protein Expression System	Protection Ratio		Advantage	Disadvantage	References
		Homologous	Heterologous			
HA stem	Eukaryotic expression	Complete protection	Partial protection; Poor protection for different HA groups	Single HA stem can produce extensive protection for the same HA group	Protection is limited by group differences	[22,23,24,25,26,27,28,29,30,31,32,33]
Chimeric HA	Eukaryotic expression or rescue chimeric attenuated virus vaccine	Complete protection	Partial protection; Poor protection for different HA groups	Easy production of chimeric attenuated vaccine	Protection is limited by the type of chimeric subtype and requires multiple immunizations with different chimeric vaccines	[24,33,34,35,36,37,38,39]
NA	Eukaryotic expression	Complete protection	Partially protected or unprotected	Strong ability to protect different HA subtype strains of the same NA type	Poor protection against different NA types	[40,41,42,43,44,45]
M1, M2e, NP	Viral vectors, plasmids	Partial protection	Generates different protection according to M1, M2e and NP sequence differences	Wide range of protection without being limited by HA group differences	Not fully protected; Poor immunogenicity requires tandem or combined expression with other proteins	[39,46,47,48,49,50,51,52,53,54,55,56,57,58,59,60,61]
Epitope peptide	Chemical synthesis, prokaryotic expression, viral vectors, plasmids	Partial protection	Different protection depending on how much dominant epitope of certain flu covered	Simple, stable, easy to synthesize, non-toxic; not restricted by HA group differences	Difficult to screen for co-conserved epitopes in large influenza databases; Limited by population MHC diversity; Poor immunogenicity	[62,63,64,65,66,67,68,69,70,71]
Mosaic	Viral vector, eukaryotic expression	Complete protection	Different protection based on the subtype on which the Mosaic design is based	Strong protection and extensive protection for different clades of the same subtype	Difficult to find a sequence that covers all epitopes in the large influenza database	[72,73,74,75,76,77,78,79,80]
Nanoparticles	Gold nanoparticles, polymers, VLPs, liposomes	Different protection according to the type of antigen loaded		Efficiently improve immunogenicity; Load multiple antigens	Complex preparation process	[81,82,83,84,85,86,87,88,89,90,91,92,93,94,95]

## References

[1] Iv M.L., Dunagan M., Kurebayashi Y., Takimoto T. (2020). Key role of the influenza a virus pa gene segment in the emergence of pandemic viruses. Viruses.

[2] Sun Y., Hu Z., Zhang X., Chen M., Wang Z., Xu G., Bi Y., Tong Q., Wang M., Sun H. (2020). R195K mutation in the PA-X protein increases the virulence and transmission of influenza A virus in mammalian hosts. J. Virol..

[3] Cheung P., Ye Z., Lee T., Chen H., Chan C., Jin D. (2020). PB1-F2 protein of highly pathogenic influenza A (H7N9) virus selectively suppresses RNA-induced NLRP3 inflammasome activation through inhibition of MAVS-NLRP3 interaction. J. Leukoc. Biol..

[4] Wang Q., Liu R., Li Q., Wang F., Zhu B., Zheng M., Cui H., Wen J., Zhao G. (2019). Host cell interactome of PB1 N40 protein of H5N1 influenza A virus in chicken cells. J. Proteom..

[5] Vasin A., Temkina O., Egorov V., Klotchenko S., Plotnikova M., Kiselev O. (2014). Molecular mechanisms enhancing the proteome of influenza a viruses: An overview of recently discovered proteins. Virus Res..

[6] Klein N., Fireman B., Goddard K., Zerbo O., Asher J., Zhou J., King J., Lewis N. (2020). Vaccine effectiveness of cell-culture relative to egg-based inactivated influenza vaccine during the 2017-18 influenza season. PLoS ONE.

[7] Sarsenbayeva G., Issagulov T., Kassenov M., Abitay R., Orynbayev M., Stukova M., Pisareva M., Davlyatshin T., Lespek K., Khairullin B. (2020). Safety and immunogenicity of trivalent inactivated influenza vaccine in adults 60 years of age and older: A phase II, a randomized, comparative trial in Kazakhstan. Hum. Vaccines Immunother..

[8] Avalos L., Ferber J., Zerbo O., Naleway A., Bulkley J., Thompson M., Cragan J., Williams J., Odouli R., Kauffman T. (2020). Trivalent inactivated influenza vaccine (IIV3) during pregnancy and six-month infant development. Vaccine.

[9] Trombetta C.M., Gianchecchi E., Montomoli E. (2018). Influenza vaccines: Evaluation of the safety profile. Hum. Vaccin Immunother..

[10] Lee G., Chu K., Inn K., Moon E., Quan F. (2019). Vaccine Efficacy Induced by 2009 Pandemic H1N1 Virus-Like Particles Differs from that Induced by Split Influenza Virus. Immunol. Investig..

[11] Kim K., Lee Y., Park S., Jung Y., Lee Y., Ko E., Kim Y., Li X., Kang S. (2019). Neuraminidase expressing virus-like particle vaccine provides effective cross protection against influenza virus. Virology.

[12] Subbarao K. (2020). Live Attenuated Cold-Adapted Influenza Vaccines. Cold Spring Harbor Perspect. Med..

[13] Holzer B., Morgan S., Martini V., Sharma R., Clark B., Chiu C., Salguero F., Tchilia N.E. (2019). Immunogenicity and protective efficacy of seasonal human live attenuated cold-adapted influenza virus vaccine in pigs. Front. Immunol..

[14] Rodriguez L., Blanco-Lobo P., Reilly E., Maehigashi T., Nogales A., Smith A., Topham D., Dewhurst S., Kim B., Martínez-Sobrido L. (2019). Comparative study of the temperature sensitive, cold adapted and attenuated mutations present in the master donor viruses of the two commercial human live attenuated influenza vaccines. Viruses.

[15] Jang Y., Kim J., Byun Y., Son A., Lee J., Lee Y., Chang J., Seong B. (2018). Pan-influenza a protection by prime-boost vaccination with cold-adapted live-attenuated influenza vaccine in a mouse model. Front. Immunol..

[16] Li H., Li Q., Li B., Guo Y., Xing J., Xu Q., Liu L., Zhang J., Qi W., Jia W. (2020). Continuous reassortment of clade 2.3.4.4 H5N6 Highly pathogenetic avian influenza viruses demonstrating high risk to public health. Pathogens.

[17] Zhang Y., Xu C., Zhang H., Liu G.D., Xue C., Cao Y. (2019). Targeting hemagglutinin: Approaches for broad protection against the influenza a virus. Viruses.

[18] Saha P., Biswas M., Gupta R., Majumdar A., Mitra S., Banerjee A., Mukherjee A., Dutta S., Chawla-Sarkar M. (2020). Molecular characterization of Influenza A pandemic H1N1 viruses circulating in eastern India during 2017–2019: Antigenic diversity in comparison to the vaccine strains. Infect. Genet. Evol. J. Mol. Epidemiol. Evol. Genet. Infect. Dis..

[19] Xue K., Bloom J. (2020). Linking influenza virus evolution within and between human hosts. Virus Evol..

[20] Wille M., Holmes E. (2019). The ecology and evolution of influenza viruses. Cold Spring Harb. Perspect. Med..

[21] Zolotarova O., Budzanivska I., Leibenko L., Radchenko L., Mironenko A. (2019). Antigenic site variation in the hemagglutinin of pandemic influenza A(H1N1)pdm09 viruses between 2009–2017 in Ukraine. Pathogens.

[22] Stray S., Pittman L. (2012). Subtype- and antigenic site-specific differences in biophysical influences on evolution of influenza virus hemagglutinin. Virol. J..

[23] Chromikova V., Tan J., Aslam S., Rajabhathor A., Bermudez-Gonzalez M., Ayllon J., Simon V., García-Sastre A., Salaun B., Nachbagauer R. (2020). Activity of human serum antibodies in an influenza virus hemagglutinin stalk-based ADCC reporter assay correlates with activity in a CD107a degranulation assay. Vaccine.

[24] Krammer F., Palese P. (2019). Universal influenza virus vaccines that target the conserved hemagglutinin stalk and conserved sites in the head domain. J. Infect. Dis..

[25] Steel J., Lowen A.C., Wang T.T., Yondola M., Gao Q., Haye K., Garcia-Sastre A., Palese P. (2010). Influenza virus vaccine based on the conserved hemagglutinin stalk domain. mBio.

[26] Wohlbold T., Nachbagauer R., Margine I., Tan G., Hirsh A., Krammer F. (2015). Vaccination with soluble headless hemagglutinin protects mice from challenge with divergent influenza viruses. Vaccine.

[27] Yassine H., Boyington J., McTamney P., Wei C., Kanekiyo M., Kon G.W., Gallagher J., Wang L., Zhang Y., Joyce M. (2015). Hemagglutinin-stem nanoparticles generate heterosubtypic influenza protection. Nat. Med..

[28] Impagliazzo A., Milder F., Kuipers H., Wagner M., Zhu X., Hoffman R., van Meersbergen R., Huizingh J., Wanningen P., Verspui J.J. (2015). A stable trimeric influenza hemagglutinin stem as a broadly protective immunogen. Science.

[29] Golchin M., Moghadaszadeh M., Tavakkoli H., Ghanbarpour R., Dastmalchi S. (2018). Recombinant M2e-HA2 fusion protein induced immunity responses against intranasally administered H9N2 influenza virus. Microb. Pathog..

[30] Trucchi C., Paganino C., Amicizia D., Orsi A., Tisa V., Piazza M., Icardi G., Ansaldi F. (2019). Universal influenza virus vaccines: What needs to happen next?. Expert Opin. Biol. Ther..

[31] Estrada L.D., Schultz-Cherry S. (2019). Development of a universal influenza vaccine. J. Immunol..

[32] Boyoglu-Barnum S., Hutchinson G.B., Boyington J.C., Moin S.M., Gillespie R.A., Tsybovsky Y., Stephens T., Vaile J.R., Lederhofer J., Corbett K.S. (2020). Glycan repositioning of influenza hemagglutinin stem facilitates the elicitation of protective cross-group antibody responses. Nat. Commun..

[33] Bajic G., Maron M., Adachi Y., Onodera T., McCarthy K., McGee C., Sempowski G., Takahashi Y., Kelsoe G., Kuraoka M. (2019). Influenza antigen engineering focuses immune responses to a subdominant but broadly protective viral Epitope. Cell Host Microbe.

[34] Broecker F., Liu S., Suntronwong N., Sun W., Bailey M., Nachbagauer R., Krammer F., Palese P. (2019). A mosaic hemagglutinin-based influenza virus vaccine candidate protects mice from challenge with divergent H3N2 strains. NPJ Vaccines.

[35] Bernstein D., Guptill J., Naficy A., Nachbagauer R., Berlanda-Scorza F., Feser J., Wilson P., Solórzano A., van der Wielen M., Walter E. (2020). Immunogenicity of chimeric haemagglutinin-based, universal influenza virus vaccine candidates: Interim results of a randomised, placebo-controlled, phase 1 clinical trial. Lancet Infect. Dis..

[36] Asthagiri Arunkumar G., McMahon M., Pavot V., Aramouni M., Ioannou A., Lambe T., Gilbert S., Krammer F. (2019). Vaccination with viral vectors expressing NP, M1 and chimeric hemagglutinin induces broad protection against influenza virus challenge in mice. Vaccine.

[37] Liu W., Nachbagauer R., Stadlbauer D., Solórzano A., Berlanda-Scorza F., García-Sastre A., Palese P., Krammer F., Albrecht R. (2019). Sequential immunization with live-attenuated chimeric hemagglutinin-based vaccines confers heterosubtypic immunity against influenza a viruses in a preclinical ferret model. Front. Immunol..

[38] McMahon M., Asthagiri Arunkumar G., Liu W., Stadlbauer D., Albrecht R., Pavot V., Aramouni M., Lambe T., Gilbert S., Krammer F. (2019). Vaccination with viral vectors expressing chimeric hemagglutinin, NP and M1 antigens protects ferrets against influenza virus challenge. Front. Immunol..

[39] Sun W., Zheng A., Miller R., Krammer F., Palese P. (2019). An inactivated influenza virus vaccine approach to targeting the conserved hemagglutinin stalk and M2e domains. Vaccines.

[40] Gubareva L., Mohan T. (2020). Antivirals Targeting the Neuraminidase. Cold Spring Harb. Perspect. Med..

[41] Abed Y., Schibler M., Checkmahomed L., Carbonneau J., Venable M., Fage C., Giannotti F., Goncalves A., Kaiser L., Boivin G. (2020). Molecular pathway of influenza pan-neuraminidase inhibitor resistance in an immunocompromised patient. Antivir. Ther..

[42] Lampejo T. (2020). Influenza and antiviral resistance: An overview. Eur. J. Clin. Microbiol. Infect. Dis..

[43] Wohlbold T., Nachbagauer R., Xu H., Tan G., Hirsh A., Brokstad K., Cox R., Palese P., Krammer F. (2015). Vaccination with adjuvanted recombinant neuraminidase induces broad heterologous, but not heterosubtypic, cross-protection against influenza virus infection in mice. mBio.

[44] Stadlbauer D., Zhu X., McMahon M., Turner J., Wohlbold T., Schmitz A., Strohmeier S., Yu W., Nachbagauer R., Mudd P. (2019). Broadly protective human antibodies that target the active site of influenza virus neuraminidase. Science.

[45] Piepenbrink M., Nogales A., Basu M., Fucile C., Liesveld J., Keefer M., Rosenberg A., Martinez-Sobrido L., Kobie J. (2019). Broad and protective influenza B virus neuraminidase antibodies in humans after vaccination and their clonal persistence as plasma cells. mBio.

[46] Ong H., Yong C., Tan W., Yeap S., Omar A., Razak M., Ho K. (2019). An influenza a vaccine based on the extracellular domain of matrix 2 protein protects BALB/C mice against H1N1 and H3N2. Vaccines.

[47] Antrobus R.D., Berthoud T.K., Mullarkey C.E., Hoschler K., Coughlan L., Zambon M., Hill A.V., Gilbert S.C. (2014). Coadministration of seasonal influenza vaccine and MVA-NP+M1 simultaneously achieves potent humoral and cell-mediated responses. Mol. Ther..

[48] Goodman A., Heinen P., Guerra S., Vijayan A., Sorzano C., Gomez C., Esteban M. (2011). A human multi-epitope recombinant vaccinia virus as a universal T cell vaccine candidate against influenza virus. PLoS ONE.

[49] Kim J., Cheong S., Lee M. (2019). Evaluation of protective immunity of peptide vaccines composed of a 15-mer N-terminal matrix protein 2 and a helper t-cell epitope derived from influenza a virus. Immune Netw..

[50] Blokhina E., Mardanova E., Stepanova L., Tsybalova L., Ravin N. (2020). Plant-produced recombinant influenza a virus candidate vaccine based on flagellin linked to conservative fragments of M2 protein and hemagglutintin. Plants.

[51] Bimler L., Song A., Le D., Murphy S.A., Paust S. (2019). AuNP-M2e+sCpG vaccination of juvenile mice generates lifelong protective immunity to influenza a virus infection. Immun. Ageing.

[52] Li L., Ren Z., Wang Q., Ge S., Liu Y., Liu C., Liu F., Hu Y., Li J., Bao J. (2019). Infection of African swine fever in wild boar, China, 2018. Transbound Emerg. Dis..

[53] Lee S., Kang J., Chang J. (2019). Nucleoprotein vaccine induces cross-protective cytotoxic T lymphocytes against both lineages of influenza B virus. Clin. Exp. Vaccine Res..

[54] Kim M., Kang J., Kim J., Jung H., Lee H., Chang J. (2019). Single mucosal vaccination targeting nucleoprotein provides broad protection against two lineages of influenza B virus. Antivir. Res..

[55] Antrobus R.D., Coughlan L., Berthoud T.K., Dicks M.D., Hill A.V., Lambe T., Gilbert S.C. (2014). Clinical assessment of a novel recombinant simian adenovirus ChAdOx1 as a vectored vaccine expressing conserved influenza A antigens. Mol. Ther..

[56] Coughlan L., Sridhar S., Payne R., Edmans M., Milicic A., Venkatraman N., Lugonja B., Clifton L., Qi C., Folegatti P.M. (2018). Heterologous two-dose vaccination with simian adenovirus and poxvirus vectors elicits long-lasting cellular immunity to influenza virus a in healthy adults. EBioMedicine.

[57] Del Campo J., Pizzorno A., Djebali S., Bouley J., Haller M., Perez-Vargas J., Lina B., Boivin G., Hamelin M.E., Nicolas F. (2019). OVX836 a recombinant nucleoprotein vaccine inducing cellular responses and protective efficacy against multiple influenza A subtypes. NPJ Vaccines.

[58] Zhao D., Liu R., Zhang X., Li F., Wang J., Zhang J., Liu X., Wang L., Zhang J., Wu X. (2019). Replication and virulence in pigs of the first African swine fever virus isolated in China. Emerg. Microbes Infect..

[59] Rowell J., Lo C., Price G., Misplon J., Crim R., Jayanti P., Beeler J., Epstein S. (2019). The effect of respiratory viruses on immunogenicity and protection induced by a candidate universal influenza vaccine in mice. PLoS ONE.

[60] Folegatti P.M., Bellamy D., Flaxman A., Mair C., Ellis C., Ramon R.L., Ramos Lopez F., Mitton C., Baker M., Poulton I. (2019). Safety and Immunogenicity of the heterosubtypic influenza a vaccine MVA-NP+M1 manufactured on the AGE1.CR.pIX avian cell line. Vaccines.

[61] Swayze H., Allen J., Folegatti P., Yu L.M., Gilbert S., Hill A., Ellis C., Butler C.C. (2019). A phase IIb study to determine the safety and efficacy of candidate INfluenza Vaccine MVA-NP+M1 in combination with licensed Ina CTivated infl Uenza vaccine in adult S aged 65 years and above (INVICTUS): A study protocol. F1000Res.

[62] Tan L., Lu H., Zhang D., Tian M., Hu B., Wang Z., Jin N. (2010). Protection against H1N1 influenza challenge by a DNA vaccine expressing H3/H1 subtype hemagglutinin combined with MHC class II-restricted epitopes. Virology J..

[63] Eickhoff C., Terry F., Peng L., Meza K., Sakala I., Van A.D., Moise L., Martin W., Schriewer J., Buller R. (2019). Highly conserved influenza T cell epitopes induce broadly protective immunity. Vaccine.

[64] Hassan A., Amen O., Sayedahmed E., Vemula S., Amoah S., York I., Gangappa S., Sambhara S., Mittal S. (2017). Adenovirus vector-based multi-epitope vaccine provides partial protection against H5, H7, and H9 avian influenza viruses. PLoS ONE.

[65] Atsmon J., Caraco Y., Ziv-Sefer S., Shaikevich D., Abramov E., Volokhov I., Bruzil S., Haima K.Y., Gottlieb T., Ben-Yedidia T. (2014). Priming by a novel universal influenza vaccine (Multimeric-001)-a gateway for improving immune response in the elderly population. Vaccine.

[66] Atsmon J., Kate-Ilovitz E., Shaikevich D., Singer Y., Volokhov I., Haim K.Y., Ben-Yedidia T. (2012). Safety and immunogenicity of multimeric-001--a novel universal influenza vaccine. J. Clin. Immunol..

[67] Lowell G.H., Ziv S., Bruzil S., Babecoff R., Ben-Yedidia T. (2017). Back to the future: Immunization with M-001 prior to trivalent influenza vaccine in 2011/12 enhanced protective immune responses against 2014/15 epidemic strain. Vaccine.

[68] Van Doorn E., Liu H., Ben-Yedidia T., Hassin S., Visontai I., Norley S., Frijlink H.W., Hak E. (2017). Evaluating the immunogenicity and safety of a BiondVax-developed universal influenza vaccine (Multimeric-001) either as a standalone vaccine or as a primer to H5N1 influenza vaccine: Phase IIb study protocol. Medicine.

[69] Pleguezuelos O., Dille J., de Groen S., Oftung F., Niesters H.G.M., Islam M.A., Næss L.M., Hungnes O., Aldarij N., Idema D.L. (2020). Immunogenicity, safety, and efficacy of a standalone universal influenza vaccine, FLU-v, in healthy adults: A randomized clinical trial. Ann. Intern. Med..

[70] Pleguezuelos O., James E., Fernandez A., Lopes V., Rosas L.A., Cervantes-Medina A., Cleath J., Edwards K., Neitzey D., Gu W. (2020). Efficacy of FLU-v, a broad-spectrum influenza vaccine, in a randomized phase IIb human influenza challenge study. NPJ Vaccines.

[71] Van Doorn E., Pleguezuelos O., Liu H., Fernandez A., Bannister R., Stoloff G., Oftung F., Norley S., Huckriede A., Frijlink H.W. (2017). Evaluation of the immunogenicity and safety of different doses and formulations of a broad spectrum influenza vaccine (FLU-v) developed by SEEK: Study protocol for a single-center, randomized, double-blind and placebo-controlled clinical phase IIb trial. BMC Infect. Dis..

[72] Stephenson K., Wegmann F., Tomaka F., Walsh S., Tan C., Lavreys L., Ansel J., Kanjilal D., Jaegle K., Nkolola J. (2020). Comparison of shortened mosaic HIV-1 vaccine schedules: A randomised, double-blind, placebo-controlled phase 1 trial (IPCAVD010/HPX1002) and a preclinical study in rhesus monkeys (NHP 17-22). Lancet HIV.

[73] Zou C., Murakoshi H., Kuse N., Akahoshi T., Chikata T., Gatanaga H., Oka S., Hanke T., Takiguchi M. (2019). Effective suppression of HIV-1 replication by cytotoxic T lymphocytes specific for pol epitopes in conserved mosaic vaccine immunogens. J. Virol..

[74] Mega E. (2019). Mosaic’ HIV vaccine to be tested in thousands of people across the world. Nature.

[75] Kamlangdee A., Kingstad-Bakke B., Osorio J. (2016). Mosaic H5 hemagglutinin provides broad humoral and cellular immune responses against influenza viruses. J. Virol..

[76] Florek N., Kamlangdee A., Mutschler J., Kingstad-Bakke B., Schultz-Darken N., Broman K., Osorio J., Friedrich T. (2017). A modified vaccinia Ankara vaccine vector expressing a mosaic H5 hemagglutinin reduces viral shedding in rhesus macaques. PLoS ONE.

[77] Kamlangdee A., Kingstad-Bakke B., Anderson T.K., Goldberg T.L., Osorio J.E. (2014). Broad protection against avian influenza virus by using a modified vaccinia Ankara virus expressing a mosaic hemagglutinin gene. J. Virol..

[78] Corder B., Bullard B., DeBeauchamp J., Ilyushina N., Webby R., Weaver E. (2019). Influenza H1 mosaic hemagglutinin vaccine induces broad immunity and protection in mice. Vaccines.

[79] Sun W., Kirkpatrick E., Ermler M., Nachbagauer R., Broecker F., Krammer F., Palese P. (2019). Development of Influenza b universal vaccine candidates using the "Mosaic" hemagglutinin approach. J. Virol..

[80] Ross T., DiNapoli J., Giel-Moloney M., Bloom C., Bertran K., Balzli C., Strugnell T., Sá E Silva M., Mebatsion T., Bublot M. (2019). A computationally designed H5 antigen shows immunological breadth of coverage and protects against drifting avian strains. Vaccine.

[81] Qi M., Zhang X., Sun X., Zhang X., Yao Y., Liu S., Chen Z., Li W., Zhang Z., Chen J. (2018). Intranasal nanovaccine confers homo- and hetero-subtypic influenza protection. Small.

[82] Ni Y., Guo J., Turner D., Tizard I. (2017). Development of a novel dual-domain nanoparticle antigen construct for universal influenza vaccine. Vaccine.

[83] Biswas A., Chakrabarti A.K., Dutta S. (2020). Current challenges: From the path of "original antigenic sin" towards the development of universal flu vaccines. Int. Rev. Immunol..

[84] Sulczewski F., Liszbinski R., Romão P., Rodrigues Junior L. (2018). Nanoparticle vaccines against viral infections. Arch. Virol..

[85] Wang C., Zhu W., Luo Y., Wang B. (2018). Gold nanoparticles conjugating recombinant influenza hemagglutinin trimers and flagellin enhanced mucosal cellular immunity. Nanomed. Nanotechnol. Biol. Med..

[86] Portnoff A., Patel N., Massare M., Zhou H., Tian J., Zhou B., Shinde V., Glenn G., Smith G. (2020). Influenza hemagglutinin nanoparticle vaccine elicits broadly neutralizing antibodies against structurally distinct domains of H3N2 HA. Vaccines.

[87] Wang Y., Deng L., Gonzalez G.X., Luthra L., Dong C., Ma Y., Zou J., Kang S.-M., Wang B.-Z. (2020). Double-layered M2e-NA protein nanoparticle immunization induces broad cross-protection against different influenza viruses in mice. Adv. Healthc. Mater..

[88] Georgiev I., Joyce M., Chen R., Leung K., McKee K., Druz A., Van Galen J., Kanekiyo M., Tsybovsky Y., Yang E. (2018). Two-Component ferritin nanoparticles for multimerization of diverse trimeric antigens. ACS Infect. Dis..

[89] Deng L., Wang B. (2018). A perspective on nanoparticle universal influenza vaccines. ACS Infect. Dis..

[90] Mezhenskaya D., Isakova-Sivak I., Rudenko L. (2019). M2e-based universal influenza vaccines: A historical overview and new approaches to development. J. Biomed. Sci..

[91] Kanekiyo M., Joyce M., Gillespie R., Gallagher J., Andrews S., Yassine H., Wheatley A., Fisher B., Ambrozak D., Creanga A. (2019). Mosaic nanoparticle display of diverse influenza virus hemagglutinins elicits broad B cell responses. Nat. Immunol..

[92] Bernasconi V., Bernocchi B., Ye L., Lê M., Omokanye A., Carpentier R., Schön K., Saelens X., Staeheli P., Betbeder D. (2018). Porous nanoparticles with self-adjuvanting M2e-fusion protein and recombinant hemagglutinin provide strong and broadly protective immunity against influenza virus infections. Front. Immunol..

[93] Adler-Moore J., Ernst W., Kim H., Ward N., Chiang S., Do T., Fujii G. (2017). Monomeric M2e antigen in VesiVax liposomes stimulates protection against type a strains of influenza comparable to liposomes with multimeric forms of M2e. J. Liposome Res..

[94] Dhakal S., Cheng X., Salcido J., Renu S., Bondra K., Lakshmanappa Y., Misch C., Ghimire S., Feliciano-Ruiz N., Hogshead B. (2018). Liposomal nanoparticle-based conserved peptide influenza vaccine and monosodium urate crystal adjuvant elicit protective immune response in pigs. Int. J. Nanomed..

[95] Wang J., Li P., Yu Y., Fu Y., Jiang H., Lu M., Sun Z., Jiang S., Lu L., Wu M.X. (2020). Pulmonary surfactant–biomimetic nanoparticles potentiate heterosubtypic influenza immunity. Science.

[96] Awate S., Babiuk L.A., Mutwiri G. (2013). Mechanisms of action of adjuvants. Front. Immunol..

[97] Uddowla S., Freytag L.C., Clements J.D. (2007). Effect of adjuvants and route of immunizations on the immune response to recombinant plague antigens. Vaccine.

[98] Renu S., Feliciano-Ruiz N., Ghimire S., Han Y., Schrock J., Dhakal S., Patil V., Krakowka S., Renukaradhya G. (2020). Poly(I:C) augments inactivated influenza virus-chitosan nanovaccine induced cell mediated immune response in pigs vaccinated intranasally. Vet. Microbiol..

[99] Schussek S., Bernasconi V., Mattsson J., Wenzel U., Strömberg A., Gribonika I., Schön K., Lycke N. (2020). The CTA1-DD adjuvant strongly potentiates follicular dendritic cell function and germinal center formation, which results in improved neonatal immunization. Mucosal Immunol..

[100] Zhou Y., Li S., Bi S., Li N., Bi Y., Liu W., Wang B. (2020). Long-lasting protective immunity against H7N9 infection is induced by intramuscular or CpG-adjuvanted intranasal immunization with the split H7N9 vaccine. Int. Immunopharmacol..

[101] Mathew M., Virmani N., Bera B., Anand T., Kumar R., Balena V., Sansanwal R., Pavulraj S., Sundaram K., Virmani M. (2019). Protective efficacy of inactivated reverse genetics based equine influenza vaccine candidate adjuvanted with montanide pet gel in murine model. J. Vet. Med Sci..

[102] Short K., Miller S., Walsh L., Cybulski V., Bazin H., Evans J., Burkhart D. (2019). Co-encapsulation of synthetic lipidated TLR4 and TLR7/8 agonists in the liposomal bilayer results in a rapid, synergistic enhancement of vaccine-mediated humoral immunity. J. Control. Release.

[103] Saelens X. (2019). The role of matrix protein 2 ectodomain in the development of universal influenza vaccines. J. Infect. Dis..

[104] Rudicell R., Garinot M., Kanekiyo M., Kamp H., Swanson K., Chou T., Dai S., Bedel O., Simard D., Gillespie R. (2019). Comparison of adjuvants to optimize influenza neutralizing antibody responses. Vaccine.

[105] Reed S.G., Orr M.T., Fox C.B. (2013). Key roles of adjuvants in modern vaccines. Nat. Med..

